# Arthroscopic Subacromial Decompression in the Treatment of Shoulder Impingement Syndrome: A Prospective Study in Malaysia

**DOI:** 10.7759/cureus.19254

**Published:** 2021-11-04

**Authors:** Khairul Nizam Siron, Muhamad Taufik Mat Lani, Chooi Leng Low, Ren Yi Kow

**Affiliations:** 1 Department of Orthopaedics, Traumatology & Rehabilitation, International Islamic University Malaysia, Kuantan, MYS; 2 Department of Radiology, International Islamic University Malaysia, Kuantan, MYS

**Keywords:** shoulder pain, malaysia, subacromial decompression, arthroscopic shoulder surgery, shoulder impingement syndrome

## Abstract

Introduction

Shoulder impingement syndrome (SIS) is one of the common problems which lead to shoulder disabilities. This condition has been described as impingement to the rotator cuff by the anterior third of the acromion process and has been classified into three stages. Treatment option varies depending on the grade of the disease. Arthroscopic subacromial decompression (ASAD) has become more popular in recent years and has shown to have a good outcome. The purpose of this study is to evaluate the outcomes following ASAD in terms of the functional, clinical, and radiological parameters in treating SIS in the ageing population in Kuantan, Pahang, Malaysia.

Materials and methods

This was an observational study looking at the outcomes of patients with stage 2 and stage 3 (partial cuff tear) impingement syndrome who underwent ASAD in Hospital Tengku Ampuan Afzan and International Islamic University Malaysia Medical Centre from May 2018 to June 2019. The functional outcomes were evaluated using American Shoulder and Elbow Surgeons (ASES) score taken at pre-operative, six weeks, three months, and six months post-operation. Clinical outcomes were evaluated using Constant score (CS) taken at six months post-operation. Radiological outcomes were measured by comparing acromiohumeral distance pre- and post-operation on anteroposterior (AP) view radiograph of the affected shoulder.

Results

A total of 28 patients were selected for the study. On functional outcome, there was a significant effect of time on the ASES scoring system (p-value <0.05) from pre-operative to six months post-operation. On clinical outcome, the CS at six months showed 13 patients have excellent scores, 10 have good, and five have fair scores. There was a statistically significant difference in mean values of all categories (p-value <0.05). In terms of the radiological outcome, this study observed a significant increase in patients’ subacromial space on X-ray from the pre-operative and post-operative treatment stages. In this study, we also observed that there was no significant difference in outcomes between partial and intact rotator cuff (RC) tears at six-month post-operation.

Conclusion

In this study, ASAD was found to be a beneficial intervention in the treatment of patients with shoulder impingement evidenced by the significant outcomes in terms of functional, clinical, and radiological parameters.

## Introduction

Shoulder impingement syndrome (SIS), a leading cause of shoulder disability, constitutes a major health problem in adults [1]. Up to 65% of all shoulder pathologies are associated with SIS, a problem causing shoulder disability, pain, and loss of function [1]. The shoulder impingement syndrome is a progressive, degenerative disease of the rotator cuff, with entrapment of the soft tissue [2]. Initially, the aetiology of SIS revolves around the extrinsic mechanism, where the reduction of subacromial space distance leads to the shoulder “impingement”. As our understanding of SIS improves, it is proposed that intrinsic factors and programmed cell death may play an equally important role in the pathogenesis of degenerative cuff tendinopathy [2,3].

SIS is mainly a clinical diagnosis and imaging modalities act as an adjunct to identify the pathology and rule out other causes of shoulder pain [3]. A plain radiograph is a useful guide as a narrowed acromiohumeral distance is a sign of rotator cuff tendinopathy or tear. Magnetic resonance imaging (MRI) and computed tomography (CT) arthrography can evaluate the rotator cuff tendon succinctly [3].

Thus far, there is no specific gold standard treatment for SIS and the treatment is often based on the surgeon's preference and is patient-specific. Many treatment options have been described in the literature, ranging from conservative to surgical interventions such as mini-open or arthroscopic, with or without cuff repair, and with or without subacromial decompression [3,4]. Historically, Neer advocates the open anterior acromioplasty, a technique in which the coracoclavicular ligament is excised with an open method [2]. With the advancing technologies and instruments enhancement, the arthroscopic technique has gained popularity among the surgeons treating SIS. Arthroscopic acromioplasty or more commonly called arthroscopic subacromial decompression (ASAD) has shown to have a good outcome and is comparable to open techniques in treating SIS [4-6]. Despite that, there is no reported study of ASAD on the Malaysian population with SIS. We aim to conduct a prospective, multi-centre study to evaluate the safety, functional, and radiological outcomes of arthroscopic subacromial decompression for shoulder impingement syndrome in Kuantan, Pahang, Malaysia.

## Materials and methods

This was a prospective study carried out in two centres in Kuantan, Pahang, Malaysia, namely, Hospital Tengku Ampuan Afzan (HTAA) and Sultan Ahmad Shah Medical Centre (SASMEC at International Islamic University Malaysia Medical Centre [IIUMMC]) from 1st of May 2018 to 1st of June 2019. The Medical Research and Ethics Committee (MREC), Ministry of Health Malaysia (MOH) has provided ethical approval for this study (NMRR-18-3527-41207). The inclusion and exclusion criteria are summarized in Table 1.

**Table 1:** The inclusion and exclusion criteria of this study. SIS, shoulder impingement syndrome; RC, rotator cuff.

Inclusion criteria
Age more than 18 years old
Patient diagnosed with SIS with or without rotator cuff tear (RC tear ≤5 cm)
Failed conservative management (minimal duration of three months)
Exclusion criteria
Massive rotator cuff tear (>5 cm)
History of fracture around the shoulder
Previous surgery of the shoulder
Pre-existing neurological disorder affecting the shoulder
Adhesive capsulitis without radiological sign of shoulder impingement
Patient refuses to participate in this study

All patients were assessed and diagnosed by a consultant specialized in shoulder surgery. All patients fulfilled the diagnosis of shoulder impingement syndrome via clinical and radiological evaluation. Clinically, all patients presented with shoulder pain and demonstrable impingement signs. Radiologically, all patients had reduced acromiohumeral distance with or without rotator cuff tear. Patients with massive rotator cuff tears (size >5cm) were excluded. All patients have undergone a trial of conservative management where analgesia and physiotherapy were prescribed for six weeks to three months. Written informed consent was obtained from the patients prior to study enrolment.

**Figure 1:**
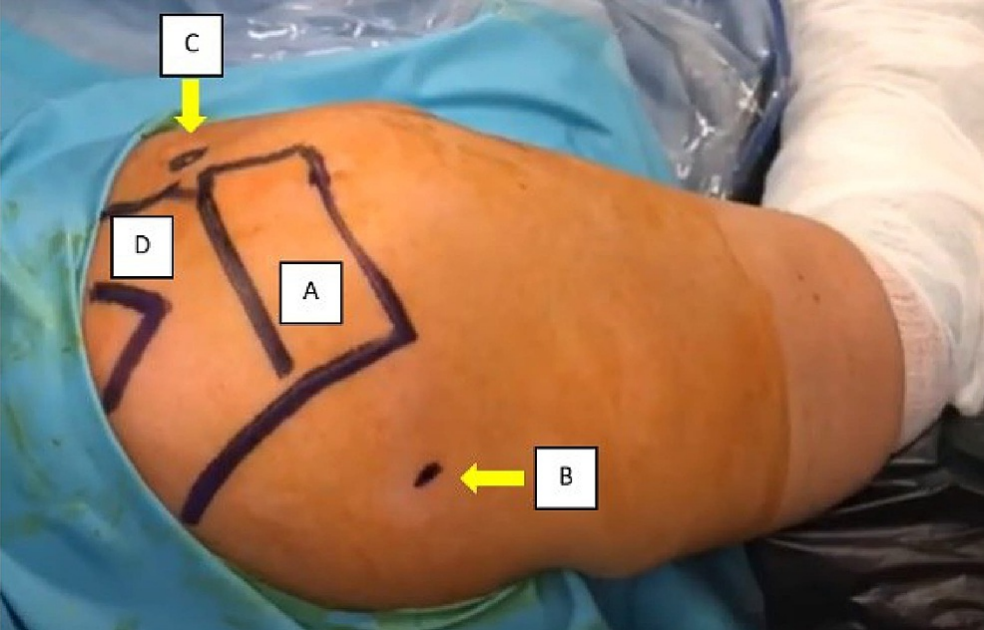
The patient was positioned in the beach chair position. The viewing portal was inserted in the posterior aspect of the shoulder by identifying the soft spot. A - acromion; B - posterior portal entry (soft spot); C - coracoid process; D - clavicle.

**Figure 2:**
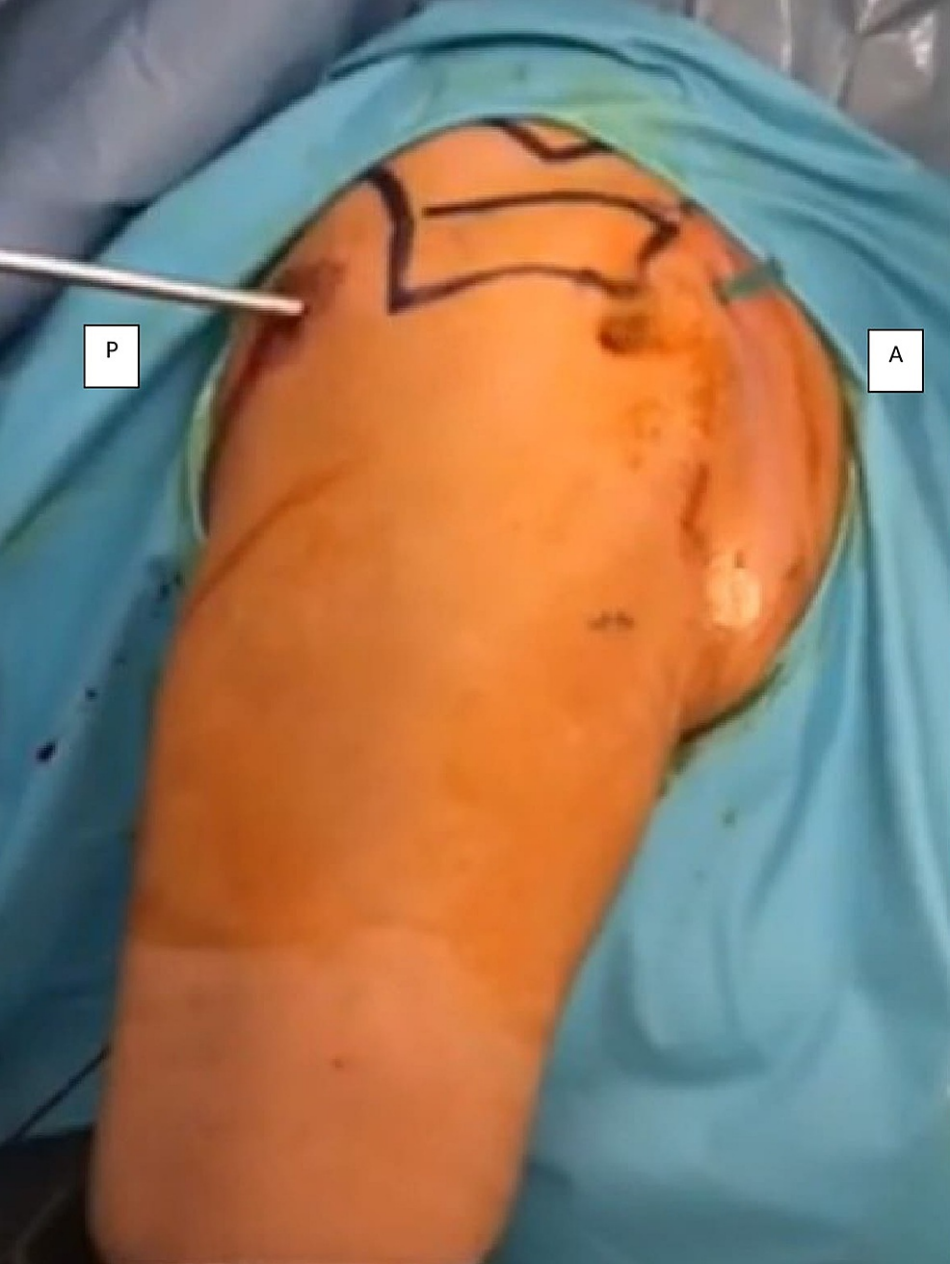
Anterior portal was identified by inserting a 21G needle at the rotator interval under arthroscopic visualization. A small incision was made for the entry of the switching stick and a dilator was inserted to facilitate the passage cannula. This was done in stages to reduce the risk of iatrogenic damage to the articular surface of the humeral head. A - anterior; P - posterior.

**Figure 3:**
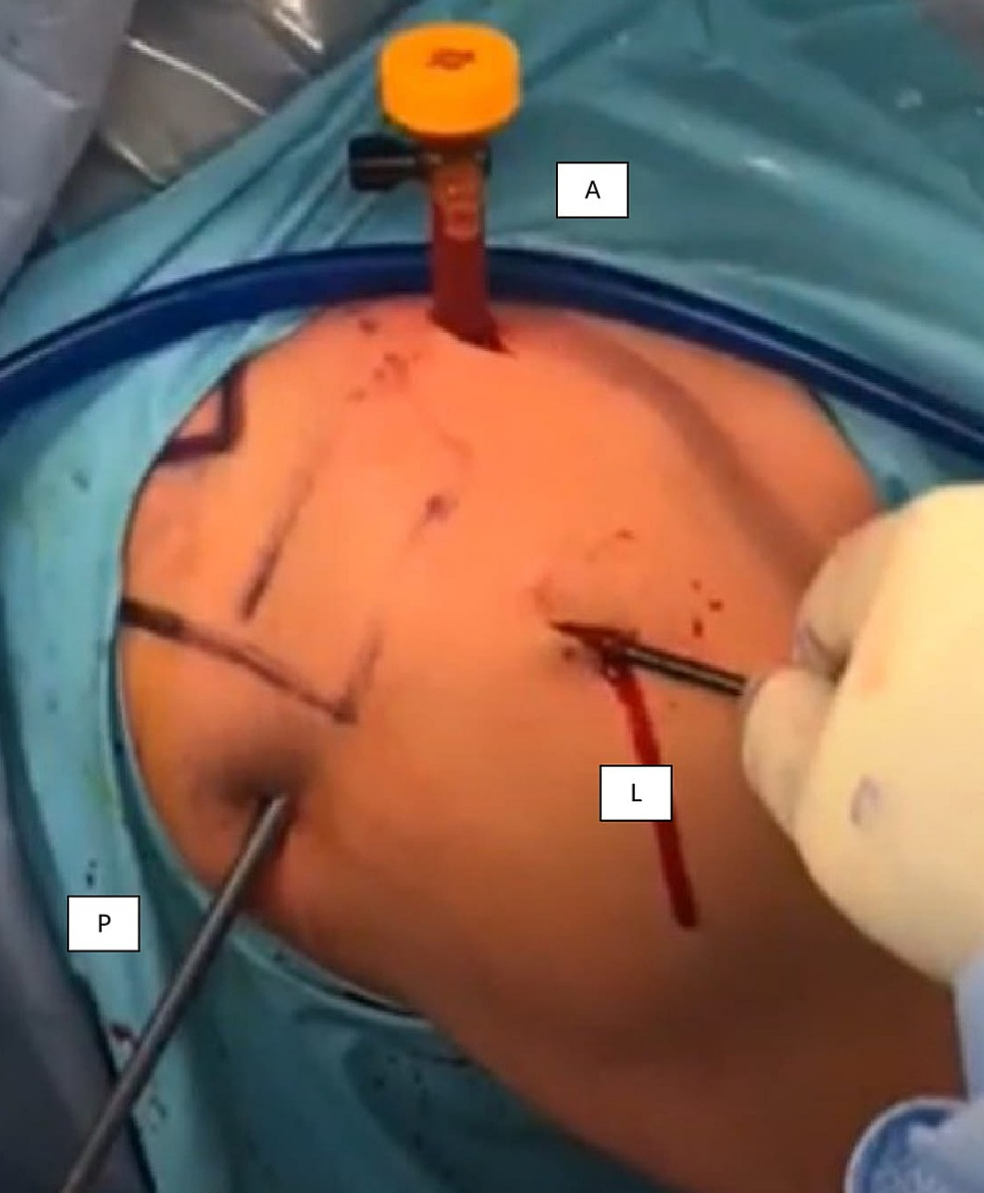
Lateral portal was identified to create a passage to the subacromial space. The location of the lateral portal was located approximately at the anterior 1/3rd of the shoulder and 2-3 cm below the acromion. A - anterior; P - posterior; L - lateral.

**Figure 4:**
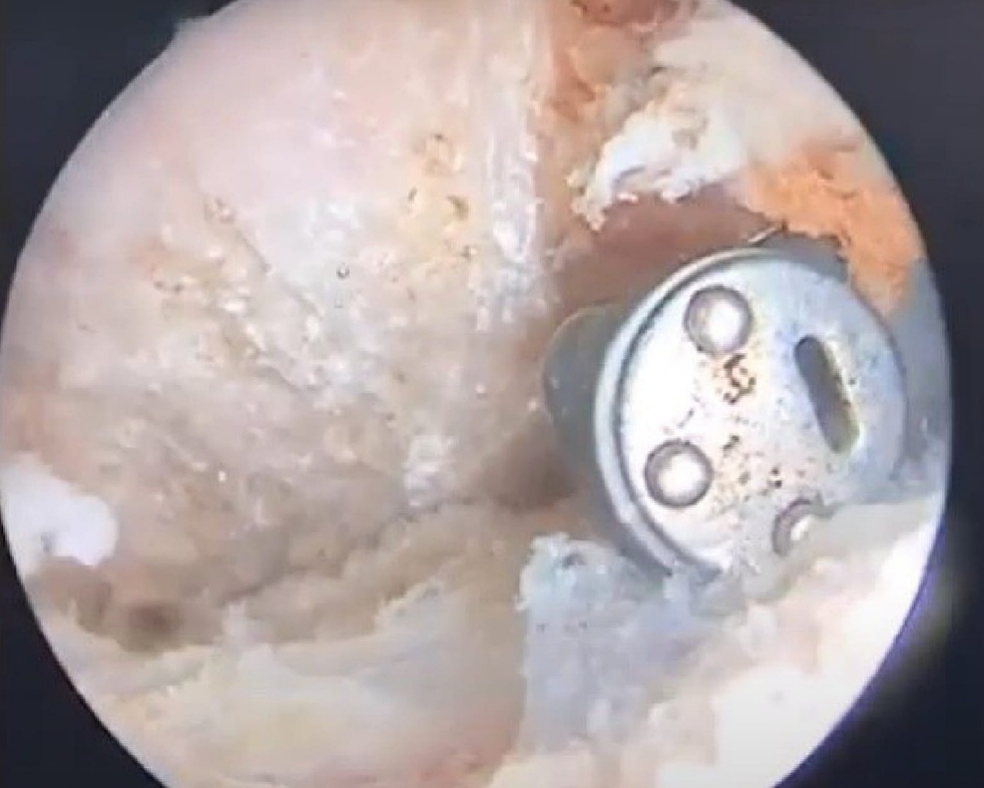
The subacromial space was debrided until the space was devoid of entrapped soft tissue.

The surgery was performed by a single surgeon to avoid inter-personnel variability. All patients had their rotator cuffs assessed intraoperatively on top of the MRI evaluation. Subacromial decompression, which includes bursal debridement and acromioplasty, was done for all patients (Figures 1-4). The partial rotator cuff tear was not repaired and was only debrided at its tear edges. All patients were started on physiotherapy involving a range of movement and strengthening exercises immediately on day one after the surgery and continued as an outpatient after discharge. As all patients did not undergo rotator cuff repair, we advocate a range of movement exercises immediately after the surgery. The patients were encouraged to abduct the shoulder up to 90 degrees for the first two weeks and a full range of movement thereafter.

The functional outcome was assessed pre-operatively, at six weeks, at three months, and at six months post-operatively with American Shoulder and Elbow Surgeons (ASES) score. The clinical outcome was assessed using a Constant score at six months post-operation in which the score was graded as excellent (score 90-100), good (80-89), fair (70-79), and poor (<70). Acromiohumeral distance from anteroposterior (AP) view of a plain radiograph of the shoulder was calculated pre- and post-operatively with a computerized system with ratio adjustment to increase the reliability. Rotator cuff conditions during intraoperative findings were also recorded.

Data collected were processed using SPSS software, version 22 (IBM SPSS Statistics, Armonk, NY). The level of p-value <0.05 was considered statistically significant. Demographic data were analysed using mean (±SD). The one-way repeated ANOVA was used to analyse the functional outcome. To evaluate the clinical outcome, one-way repeated ANOVA was used, and the result was confirmed by a post hoc analysis using the Bonferroni test. To measure the difference in acromiohumeral distance (X-ray) between pre-operative and post-operative stages (radiological outcome), the paired t-test analysis was used.

## Results

A total of 28 patients were included in this study. The mean age of the patients was 55.14 years (ranging from 43 to 70 years). The majority of the patients were female (61%). More than half of the patients had intact rotator cuff (57%). The demographic data and clinical outcomes are summarized in Table 2.

**Table 2:** Summary of the patients' demographic and outcomes. * Paired t-test; ** one-way repeated measure ANOVA test; ASES - American Shoulder and Elbow Surgeons; ASES1 - American Shoulder and Elbow Surgeons score at pre-operative; ASES2 - American Shoulder and Elbow Surgeons score at six weeks post-operative; ASES3 - American Shoulder and Elbow Surgeons score at three months post-operative; ASES4 - American Shoulder and Elbow Surgeons score at six months post-operative.

Factors		Number (n)	Percentage (%)	Mean (SD)	p-value
Age				55.14 (7.32)	
Gender	Male	11	39		
	Female	17	61		
Rotator cuff	Intact	16	57		
	Partial tear	12	43		
Acromiohumeral distance	Pre-operative			9.77 (SD 1.41)	0.001**
	Post-operative			10.28 (SD 0.94)	
Post-operative Constant score	Poor (<70)	0	0		<0.001**
	Fair (70-79)	5	17.9		
	Good (80-89)	13	35.7		
	Excellent (90-100)	10	46.4		
ASES score	ASES1			16.07 (4.91)	<0.001**
	ASES2			47.82 (10.77)	
	ASES3			67.43 (10.97)	
	ASES4			88.21 (8.76)	
Complication	Infection	None			
	Neurology deficit	None			
	Others	None			

The patients' acromiohumeral distance increased significantly after the surgical intervention, increasing from 9.77 mm to 10.28 mm (p = 0.001). Clinically, more than 80% of the patients attained good and excellent outcomes at six months post-operation in terms of Constant score. The scoring based on American Shoulder and Elbow Surgeons (ASES) criteria also showed a steadfast improvement from pre-operative (16.07) to six weeks post-operative (47.82), three months post-operative (67.43), and six months post-operative (88.21).

A one-way repeated measures ANOVA, as represented in Table 2, was conducted to compare the ASES scoring system at different time points: pre-operative, six weeks post-operative, three months post-operative, and six months post-operative. There was a significant effect of time on the ASES scoring system (Wilks’ lambda = 0.018, F (3,25) = 454.28, p < 0.001). Post hoc tests using the Bonferroni correction shows that the differences in each pair of ASES between time points were all significant (Table 3). We, therefore, conclude that the time for each ASES scoring system elicits a statistical significance over time.

**Table 3:** Comparison of ASES scoring system between different time points. * Post hoc test with Bonferroni correction; ASES - American Shoulder and Elbow Surgeons; ASES1 - American Shoulder and Elbow Surgeons at pre-operative; ASES2 - American Shoulder and Elbow Surgeons at six weeks post-operative; ASES3 - American Shoulder and Elbow Surgeons at three months post-operative; ASES4 - American Shoulder and Elbow Surgeons at six months post-operative.

ASES		Mean	Standard error	95% confidence interval		p-value*
				Lower	Upper	
Pair 1	ASES1 - ASES2	-31.75	2.09	-37.70	-25.80	<0.001
Pair 2	ASES1 - ASES3	-51.36	2.50	-58.46	-44.25	<0.001
Pair 3	ASES1 - ASES4	-72.14	1.92	-77.62	-66.67	<0.001
Pair 4	ASES2 - ASES3	-19.61	1.62	-24.22	-15.00	<0.001
Pair 5	ASES2 - ASES4	-40.39	1.85	-45.67	-35.12	<0.001
Pair 6	ASES3 - ASES4	-20.79	1.88	-26.13	-15.44	<0.001

A further sub-analysis showed the clinical outcomes are not associated with the rotator cuff integrity (p > 0.05) (Table 4).

**Table 4:** Association between rotator cuff integrity and clinical outcomes. * Independent t-test; ASES - American Shoulder and Elbow Surgeons.

Clinical outcome	Rotator cuff integrity		p-value
	Intact	Partial tear	
Mean ASES at six months post-operative (SD)	89.06 (7.73)	87.08 (10.22)	0.564*
Constant score	87.81 (6.04)	87.00 (7.83)	0.759*

## Discussion

The treatment of shoulder impingement syndrome includes both operative and non-operative approaches, and the treatment method varies considerably by the severity of the cases. Nevertheless, controversy exists in the literature concerning both operative and non-operative methods in shoulder injury treatment. Several studies argued that conservative treatments are effective for many people, especially in minor cases. For example, a study by Clausen et al. observed significant improvements in patient-reported outcomes of conservatively treated shoulder impingement patients, although they did not improve in objective clinical outcomes [7]. Likewise, Garving et al. also noted that 60% of shoulder impingement cases respond well to conservative treatment within two years [8].

While conservative non-operative treatments like physical therapy and non-steroidal anti-inflammatory drugs are found to be effective for many people, a surgical method is usually necessary when patients failed conservative treatment. Arthroscopic is one of the surgical methods which are gaining popularity in the treatment of persistent shoulder injuries. This is due to its simple surgical procedures, as well as smaller chance of infection and faster healing process compared to other open surgical methods [9]. With the case of rotator cuff tears, some recent clinical studies indicated successful results in shoulder impingement patients, with full or partial rotator cuff tears, after being treated through the arthroscopic method [10,11].

Still, it is debatable in the literature whether arthroscopic rotator cuff repair would lead to better functional and clinical outcomes in shoulder impingement patients. Ghodadra et al. (2009) supported that the all-arthroscopic approach is increasingly being adopted by surgeons as it helps to reduce patient morbidity through decreased surgical trauma [12]. A study by Attiq-ur-Rehman et al. showed that arthroscopic subacromial decompression is effective in reducing pain while improving functional outcomes of in shoulder impingement patients, regardless of the symptoms’ duration [13]. In another study, Kiran et al. concluded that the arthroscopic method has a definitive role in primary shoulder impingement [14].

In line with the above, this study sought to confirm shoulder impingement patients’ outcomes functionally and clinically after they were surgically treated through the arthroscopic method.

Functional outcomes

The ANOVA test results indicated that there was a significant difference in the ASES score between treatment groups, i.e. at different time points of the treatment. Specifically, there were notable improvements demonstrated in patients’ shoulder outcome scores (ASES score) as the time of treatment progressed from stage to stage, i.e. from the pre-operative stage to all of the post-operative stages (six weeks, three months, and six months). Moreover, post hoc tests using the Bonferroni correction also indicated a significant difference in the ASES scores between each pair of time points.

Therefore, our study supports the literature that there is a significant main effect of time on the functional outcomes among patients with surgically treated shoulder impingement syndrome, specifically the arthroscopic method. This finding is concurrent with what has been reported by Kiran et al. [14]. In their research, patients with primary shoulder impingement syndrome with arthroscopic subacromial decompression were found to have a statistically significant improvement in shoulder function after surgery compared to conservative treatment. At the 12-month post-operative stage, patients achieved good to excellent results in terms of functional outcomes following arthroscopic subacromial decompression [14].

Similarly, another study also demonstrated a significant improvement in both subjective and objective outcomes six months after arthroscopic subacromial decompression in patients with subacromial impingement syndrome who have had previous failed conservative treatment in terms of their activities of daily living following the surgery, and thus the arthroscopic method is seen as a successful treatment option for patients with subacromial impingement syndrome even with different criteria for outcome assessment [15]. Several other studies also found significant improvement in the overall mean of ASES scores post-operatively compared to the pre-operative level among patients undergoing arthroscopic subacromial decompression [16,17]. Despite this, some studies in the literature somehow noted a contrary finding. For instance, in a study by Provencher et al., uniformly lower scores were obtained by surgically treated patients compared to those managed non-operatively, regardless of the shoulder condition [18]. However, this difference could be explained by the age factor. Provencher et al.’s study findings were obtained from younger population patients with an average age of 36.5 ± 12.9 years, which might not be applicable towards the older, non-athletic patient population. Our study, on the other hand, involved older patients with an average age of 55.14 ± 7.32 years. According to Flurin et al. (2013), rotator cuff tears are more common injuries in older patients than younger groups who are more often to have labral tears [16]. Therefore, this study supported existing bodies of literature on the prevalence of shoulder pain among elderly patients as well as the potential of arthroscopic subacromial decompression for improving their functional outcome.

Clinical outcomes

The clinical outcomes at the six-month post-operative stage were evaluated using the Constant scoring system. Based on the Constant scoring system, the one-way ANOVA results showed that at this treatment stage, there was a statistically significant difference in mean values of all categories. Therefore, our finding supports that there was a significant improvement in the six-month post-operative clinical outcome of patients with shoulder impingement syndrome based on the Constant scoring system. In 2009, a similar finding was observed in a quasi-experimental study by Attiq-ur-Rehman et al. to describe the outcome of cases with subacromial impingement syndrome managed with arthroscopic subacromial decompression [13]. In their study, post-operatively significant improvement was observed in the Constant scores in all the patients, which were progressive over six months. Moreover, patients aged 40 and above as well as those with a longer duration of symptoms showed a greater improvement in shoulder scores, due to their long-standing symptoms and lower pre-operative shoulder scores [13]. Another study by Björnsson et al. in 2010 observed a similar result [19]. When evaluating the rotator cuff integrity in 70 patients 15 years after arthroscopic subacromial decompression, they found that the procedure could minimize the prevalence of rotator cuff tears in impingement patients [19]. In a similar vein, Magaji et al. who conducted a study in 2012 to assess whether arthroscopic subacromial decompression is an effective intervention in patients with symptoms for over six months due to subacromial shoulder impingement found that the treatment can be effective in selected patients, with a significant improvement in outcome scores at three months [11].

In this study, we revealed that there was no statistically significant difference in the mean values between partial and intact tears at the six-month post-operative stage based on the independent t-test. Similarly, the second analysis on the clinical outcome between Constant results and the presence of rotator cuff partial tear also indicated that there was no significant difference in the mean Constant scores between partial and intact tears. Therefore, it can be inferred from this finding that patients undergoing shoulder arthroscopy with partial tears respond to treatment as well as those with the intact rotator cuff.

A study by Lawson-Smith et al. found similar results regarding clinical outcomes of patients following arthroscopic subacromial decompression with varying sizes of bursal-side tears [20]. It was observed in their study that the size of the low-grade tear did not affect patients’ outcomes, and tears of greater than 10 mm showed similar improvements in Constant score to smaller tears and indeed cuffs with no tears. It is known that partial thickness tears have low healing ability with non-protective treatment; therefore, partial thickness tears, especially of greater size, have the possibility of becoming enlarged, and thus would cause patients to feel pain and weakness again [20]. This was not reflected in their study, as similarly observed in the present study. In a similar vein, in another study looking at the long-term outcome of arthroscopic subacromial decompression for patients with impingement syndrome, it was found that all of the patients, with or without rotator cuff tears, achieved successful results of Constant score [10].

Radiological outcomes

Based on the paired t-test analysis, it was found that there was a statistically significant difference in mean values between the two treatment stages. Specifically, the post-operative X-ray had a higher mean value than the pre-operative X-ray. This suggests that there was a significant improvement in patients’ acromiohumeral distance following arthroscopic subacromial decompression.

Several studies in the literature have shown significant radiological outcomes of the arthroscopic method for treating shoulder disorders but mostly following a rotator cuffs repair. For instance, Pepe et al. in 2018 conducted a study to evaluate the effect of the rotator cuff tear repair on subacromial space volume involving patients who were treated with shoulder arthroscopy for unilateral full-thickness small to medium rotator cuff tear and normal contralateral shoulder joint [21]. It was found in their study that the subacromial volume increase from pre-operative to post-operative was statistically significant [21].

However, in this study in which only patients with no rotator cuffs tear and partial cuffs tear were included, we have observed that 16 patients have normal (≥10mm) acromiohumeral distance in pre-operative measurement. While 11 patients have 7-9.9 mm and one patient has <7 mm acromiohumeral distance, respectively. This finding was in line with a study, which showed that the size of tendon tears and fatty muscle degeneration in the rotator cuff significantly influence the acromiohumeral distance and correlate with reduced acromiohumeral distance [22].

Based on our study findings, we observed that despite the majority of patients having normal acromiohumeral distance, there was still a significant difference in post-operative radiological outcome in comparison to pre-operative outcomes. This proved that ASAD does increase the subacromial space, resulting in improvement of the symptoms and preventing further impingement.

Implication of results

This study has provided beneficial evidence and supported the literature which put forward the important role of arthroscopic treatment in providing good to excellent functional and clinical outcomes in patients having shoulder impingement syndrome. In the light of one finding, this study suggested that functional improvement and comfort among patients after surgery for shoulder impingement syndrome can significantly increase from the pre-operative stage up to six weeks, three months, and six months post-operatively. Other than that, this study also revealed that the clinical outcome of patients with shoulder impingement syndrome has significantly improved post-operatively, regardless of the partial and intact rotator cuff tear condition. In line with a large body of evidence in the literature, this study has shown good outcomes and improved shoulder function in patients treated with the arthroscopic method. For instance, arthroscopic subacromial decompression could lead to significant improvement in function and quality of life in a cost-effective manner and it is also effective for pain reduction and improving functional outcomes of the patients [13,23].

Looking at the age factor in functional and clinical outcomes, our study also put forward that middle-aged and ageing patients should not be considered as less suitable than others for undergoing arthroscopic methods in treating their shoulder impingement syndrome. With a mean age of 55.14 (±7.32) years, patients in this study indicated significant improvement in terms of functional and clinical outcomes by ASES and Constant scores from pre-operative to six-month post-operative stages. Therefore, our study findings suggest that there may be a clinically significant benefit to consider surgical intervention in the treatment of shoulder impingement treatment, including for the ageing population. In fact, studies have also shown that the arthroscopic method consistently provides a good surgical outcome and restores working ability in varying groups of patients, such as both the workers' compensation and non-workers' compensation populations [24].

Most importantly, this study substantiates previous findings and supports the use of the ASES scoring system as a standard, reliable, and feasible method for evaluating and reporting the surgical outcome of patients with various shoulder disorders. Other than that, the Constant scoring system, which is one of the most commonly used shoulder scoring systems worldwide, is deemed to be effective in evaluating and measuring patients’ clinical outcomes following the arthroscopic method. In light of the study findings, Constant scoring was found to be useful in predicting patients’ quality of life and used in adjunct with ASES scores to assess surgical outcomes in a comprehensive manner. In addition, imaging also plays a vital role in the pre- and post-operative evaluation of shoulder disorders.

Limitations

The present study had several limitations. First, the follow-up period of the arthroscopic method in treating shoulder impingement syndrome was relatively short in this study, i.e. up until the six-month post-operative stage, compared with other clinical studies that usually followed up patients for two years after the operation. The significant difference in patients’ treatment outcomes within this short follow-up period would result in recall bias, and thus drawing conclusions by comparing our study results with those of longer year follow-up period would be quite challenging. Therefore, further studies could explore the aspect for at least one-year post-operative visit, which would have greater clinical importance due to the effect of patients’ follow-up period on long-term treatment outcomes.

Other than that, the average age of patients in the study was 55.14 years, which is considered the ageing population. Due to the fact that impingement syndrome and rotator cuff tears are common in older patients, the effect of age on patients’ functional and clinical outcomes following surgery remained under-explored in this study [16]. Moreover, the question remains as to whether their significant improvement differs by an active lifestyle. If such influence exists, results obtained from this study may not be applicable to the non-athletic ageing patient population. Therefore, it is recommended that future studies could look into different subgroups of ageing patients, such as by age ranges and involvement in physical activities.

In this prospective observational study, we did not include a control group, hence limiting the strength of the study. Furthermore, our selection criteria are more stringent than other studies, whereby we only offer surgery to patients with clinical and radiological evidence of shoulder impingement syndrome. This may explain why our results were favourable compared to other studies [25,26]. Multiple systematic reviews showed that subacromial decompression does not offer additional benefit in the treatment of patients with rotator cuff tears [25,26].

In addition, this study does not consider patients’ health-related issues, such as diabetes, which are potential factors influencing the overall recovery of patients with surgically treated shoulder impingement syndrome. As reported by several authors, patients with diabetes mellitus have a relatively poor outcome due to shoulder disorders compared to those without such conditions [27,28]. Therefore, it is recommended that samples for future studies are stratified according to the disability level to accommodate the varying health factors. Future studies could also identify and screen for patients’ psychological factors because poor psychological function could also influence patients’ pain recovery and physical ability pre-operatively and post-operatively.

## Conclusions

Overall, arthroscopic subacromial decompression was found in this study to be a beneficial intervention in the treatment of patients with shoulder disorders. The arthroscopic method was deemed to be effective in treating patients with shoulder impingement syndrome, with either partial or intact rotator cuff tears. In terms of functional outcomes, the ASES scores revealed significant improvement in patients’ functional outcomes over time. In terms of clinical outcomes as observed from the Constant scores, arthroscopic subacromial decompression has led to significant improvement in treating shoulder impingement syndrome in patients six months after their surgery. All patients undergoing shoulder arthroscopy respond to the treatment well, and this was not significantly affected by the different sizes of rotator cuff tears. In terms of radiological outcomes, this study observed a significant increase in patients’ subacromial space on X-ray from the pre-operative and post-operative treatment stages, suggesting the possible association between subacromial space volume and shoulder impingement syndrome. As an implication, this study put forward the importance of arthroscopic subacromial decompression and has provided necessary justification for its ongoing practice towards improving functional, clinical, and radiological outcomes of patients with shoulder impingement syndrome.

## References

[1] Vecchio P, Kavanagh R, Hazleman BL, King RH (1995). Shoulder pain in a community-based rheumatology clinic. Br J Rheumatol.

[2] Dong W, Goost H, Lin XB (2015). Treatments for shoulder impingement syndrome: a PRISMA systematic review and network meta-analysis. Medicine (Baltimore).

[3] Dorrestijn O, Stevens M, Winters JC, van der Meer K, Diercks RL (2009). Conservative or surgical treatment for subacromial impingement syndrome? A systematic review. J Shoulder Elbow Surg.

[4] Milano G, Grasso A, Salvatore M, Zarelli D, Deriu L, Fabbriciani C (2007). Arthroscopic rotator cuff repair with and without subacromial decompression: a prospective randomized study. Arthroscopy.

[5] Biberthaler P, Beirer M, Kirchhoff S, Braunstein V, Wiedemann E, Kirchhoff C (2013). Significant benefit for older patients after arthroscopic subacromial decompression: a long-term follow-up study. Int Orthop.

[6] Bidwai AS, Birch A, Temperley D, Odak S, Walton MJ, Haines JF, Trail I (2016). Medium- to long-term results of a randomized controlled trial to assess the efficacy of arthoscopic-subacromial decompression versus mini-open repair for the treatment of medium-sized rotator cuff tears. Shoulder Elbow.

[7] Clausen MB, Merrild MB, Witten A, Christensen KB, Zebis MK, Hölmich P, Thorborg K (2018). Conservative treatment for patients with subacromial impingement: changes in clinical core outcomes and their relation to specific rehabilitation parameters. PeerJ.

[8] Garving C, Jakob S, Bauer I, Nadjar R, Brunner UH (2017). Impingement syndrome of the shoulder. Dtsch Arztebl Int.

[9] Paxton ES, Backus J, Keener J, Brophy RH (2013). Shoulder arthroscopy: basic principles of positioning, anesthesia, and portal anatomy. J Am Acad Orthop Surg.

[10] Jaeger M, Berndt T, Rühmann O, Lerch S (2016). Patients with impingement syndrome with and without rotator cuff tears do well 20 years after arthroscopic subacromial decompression. Arthroscopy.

[11] Magaji SA, Singh HP, Pandey RK (2012). Arthroscopic subacromial decompression is effective in selected patients with shoulder impingement syndrome. J Bone Joint Surg Br.

[12] Ghodadra NS, Provencher MT, Verma NN, Wilk KE, Romeo AA (2009). Open, mini-open, and all-arthroscopic rotator cuff repair surgery: indications and implications for rehabilitation. J Orthop Sports Phys Ther.

[13] Attiq-ur-Rehman Attiq-ur-Rehman, Wajid MA, Ahmad T (2009). Shoulder impingement syndrome: outcome of arthroscopic subacromial decompression. J Coll Physicians Surg Pak.

[14] Kiran Ravi HG, Adnan Siddique P, Adarsh T, Vijay C, Mruthyunjaya Mruthyunjaya, Supreeth N (2017). Functional outcome of arthroscopic subacromial decompression in primary shoulder impingement syndrome due to extrinsic mechanical causes. Int J Orthop Sci.

[15] Bhattacharyya R, Edwards K, Wallace AW (2014). Does arthroscopic sub-acromial decompression really work for sub-acromial impingement syndrome: a cohort study. BMC Musculoskelet Disord.

[16] Flurin PH, Hardy P, Abadie P (2013). Rotator cuff tears after 70 years of age: a prospective, randomized, comparative study between decompression and arthroscopic repair in 154 patients. Orthop Traumatol Surg Res.

[17] Odak S, Powell E, Temperley D, Haines JF, Trail I (2012). A randomized controlled trial to assess the efficacy of arthroscopic subacromial decompression with and without rotator cuff repair using a mini-open technique. Shoulder Elbow.

[18] Provencher MT, Frank RM, Macian D (2012). An analysis of shoulder outcomes scores in 275 consecutive patients: disease-specific correlation across multiple shoulder conditions. Mil Med.

[19] Björnsson H, Norlin R, Knutsson A, Adolfsson L (2010). Fewer rotator cuff tears fifteen years after arthroscopic subacromial decompression. J Shoulder Elbow Surg.

[20] Lawson-Smith M, Al-Maiyah M, Goodchild L, Fourie JM, Finn P, Rangan A (2015). Do partial thickness, bursal side cuff tears affect outcome following arthroscopic subacromial decompression? A prospective comparative cohort study. Shoulder Elbow.

[21] Pepe M, Kocadal O, Gunes Z, Calisal E, Aksahin E, Aktekin CN (2018). Subacromial space volume in patients with rotator cuff tear: the effect of surgical repair. Acta Orthop Traumatol Turc.

[22] Saupe N, Pfirrmann CW, Schmid MR, Jost B, Werner CM, Zanetti M (2006). Association between rotator cuff abnormalities and reduced acromiohumeral distance. AJR Am J Roentgenol.

[23] Butt U, Whiteman A, Wilson J, Paul E, Roy B (2015). Does arthroscopic subacromial decompression improve quality of life. Ann R Coll Surg Engl.

[24] Consigliere P, Haddo O, Levy O, Sforza G (2018). Subacromial impingement syndrome: management challenges. Orthop Res Rev.

[25] Rossi LA, Ranalletta M (2020). Subacromial decompression is not beneficial for the management of rotator cuff disease. JBJS Rev.

[26] Karjalainen TV, Jain NB, Page CM (2019). Subacromial decompression surgery for rotator cuff disease. Cochrane Database Syst Rev.

[27] Burner T, Abbott D, Huber K (2014). Shoulder symptoms and function in geriatric patients. J Geriatr Phys Ther.

[28] Clement ND, Hallett A, MacDonald D, Howie C, McBirnie J (2010). Does diabetes affect outcome after arthroscopic repair of the rotator cuff?. J Bone Joint Surg Br.

